# Improvement of mild photoaged facial skin in middle‐aged Chinese females by a supramolecular retinol plus acetyl hexapeptide‐1 containing essence

**DOI:** 10.1002/ski2.239

**Published:** 2023-05-07

**Authors:** Ying Ye, Yanan Li, Chenlan Xu, Xiaolan Wei

**Affiliations:** ^1^ Research & Innovation Center Proya Cosmetics Co. Ltd. Hangzhou China

## Abstract

**Background:**

The anti‐ageing gold standard, retinol, has been widely recognized for its anti‐wrinkle benefits in the Chinese population. Studies have shown that Asians are more sensitive to retinol compared to their Caucasian counterparts, and it is generally recommended to use retinol once a day in the evening. However, there are few reports on the most appropriate concentration and frequency of retinol use in the general Chinese population.

**Objectives:**

In this study, supramolecular retinol was prepared using cyclodextrin encapsulation technology, and the most appropriate concentration for the general Chinese population was investigated. Then, a cosmetic essence was developed by combining the classic supramolecular retinol, which promotes collagen regeneration, with acetyl hexapeptide‐1, a popular ingredient known for reducing expression lines. The safety and efficacy of this cosmetic essence were studied through clinical tests.

**Methods:**

First, a patch test was conducted on 32 healthy Chinese subjects to compare the tolerance of supramolecular retinol to non‐encapsulated retinol and to select the optimal concentration of retinol. Then, an 8‐week clinical study was conducted using a twice‐daily cosmetic essence containing 0.1% supramolecular retinol and 0.02% acetyl hexapeptide‐1 to treat mild photoaging in 32 middle‐aged Chinese women. Dermatological evaluations and instrument measurements were taken at baseline, 4 weeks, and 8 weeks. Efficacy was assessed using facial skin wrinkles, textures, elasticity, firmness, pores, gloss and stratum corneum hydration. Tolerability was assessed throughout the study.

**Results:**

Our patch test results showed that supramolecular retinol was better tolerated than non‐encapsulated retinol, and our findings suggest that 0.1% was the approximate optimal retinol concentration for the general Chinese population. The cosmetic essence studied was effective in improving the appearance of photoaged skin in the Chinese population in all aspects studied and was well tolerated.

**Conclusions:**

0.1% retinol is suitable for twice daily use in the general Chinese population. Data and records on efficacy dimensions of skin textures, elasticity, firmness, pores, gloss and stratum corneum hydration for retinol in the Chinese population are supplemented with our study. Cosmeceutical approaches targeting both static and dynamic wrinkles are of value for treating the photoaged Chinese population.

## Graphical abstract

(1) Our findings suggest 0.1% is the approximate optimal retinol concentration in the general Chinese population. (2) Data and records on efficacy dimensions of skin textures, elasticity, firmness, pores, gloss and stratum corneum hydration for retinol in Chinese population are supplemented with our study. (3) Cosmeceutical approaches targeting both static and dynamic wrinkles are of value to treat photoaged Chinese population.

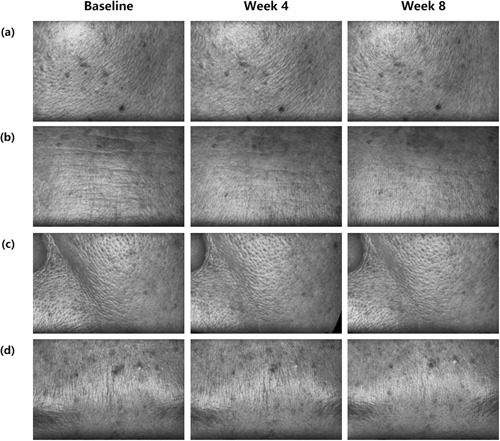

**What is already known about this topic?**

- Studies have shown that Asians are more sensitive to retinol compared to their Caucasian counterparts.
- At present, the clinical studies on topical retinol in Chinese population mainly report the anti‐wrinkle effect.
- Retinol and acetyl hexapeptide‐1 are popular ingredients for static and dynamic wrinkles, respectively.

**What does this study add?**

- Our findings suggest 0.1% is the approximate optimal retinol concentration in the general Chinese population.
- Data and records on efficacy dimensions of skin gloss, pore fineness, elasticity, firmness, roughness, smoothness and stratum corneum hydration for retinol in Chinese populations are supplemented with our study.
- Cosmeceutical approaches targeting both static and dynamic wrinkles are of value to treat photoaged Chinese population.

## 1 INTRODUCTION

In recent years, with the rise of ingredient‐conscious consumers, the anti‐ageing gold standard retinol has received widespread attention from consumers all over the world, including China. In the Caucasian population, some scholars have studied the improvement effect and mechanism of retinol on naturally aged skin,^1^ the parallel study of the efficacy of different concentrations of retinol^2^ and the retinol efficacy rate.^3^ There are relatively few studies on the efficacy of retinol in Asian populations. Some scholars have performed a comparative study of 0.04% versus 0.075% retinol using a once‐nightly regimen to treat mild photoaging in middle‐aged Japanese females.^4^ Their efficacy study has focussed on wrinkle reducing effect. Results have shown that a 0.04% retinol cream reveals less prominent improvements than 0.075% retinol in fine wrinkling but minimal irritation.

Products with retinol as the active ingredient are anti‐ageing topical products recognized by the dermatology community and cosmetic industry. However, the use of retinol may cause certain adverse reactions, such as dryness, erythema and desquamation. These adverse reactions greatly limit the use of retinol products.^5^, ^6^ Studies have shown that Asians are more sensitive to retinol compared to their Caucasian counterparts,^7^ and it is generally recommended to use this product once a day in the evening. However, there are few reports on the most appropriate concentration and frequency of retinol use in the general Chinese population.

In this study, we conducted a series of patch tests in the Chinese population to determine if the cyclodextrin encapsulation technique could relieve retinol‐induced irritation and to investigate the most appropriate retinol concentration based on safety concerns. Aiming at the problem of intolerance of retinol in the Chinese population, supramolecular retinol obtained by cyclodextrin encapsulation technology was developed.^8^, ^9^ We performed 24‐h patch tests to verify that supramolecular retinol was milder than non‐encapsulated retinol at various test concentrations (0.05%, 0.1%, 0.2%, 0.3%, 0.5%). Moreover, our patch test results indicated that 0.1% retinol is the approximate optimal concentration for the general Chinese population, which balances efficacy and safety.

Then, we developed an essence formulated with 0.1% supramolecular retinol and 0.02% acetyl hexapeptide‐1, and we carried out an 8‐week clinical trial using the essence twice daily to study the effect of the essence on the skin of a mildly photoaged Chinese population with an average age of 49.5 years. The essence design concept was to promote collagen and glucosamine regeneration through supramolecular retinol to improve permanent wrinkles; inhibit neurotransmitters through acetyl hexapeptide‐1 to improve expression lines and achieve a safer and more effective active ingredient combination with a synergetic mechanism of action. According to recent market trends, consumers are increasingly expecting products with higher efficacy to quickly beautify and improve skin condition. Both the dermatologist community and the cosmetic industry hope to address functional issues, improve skin appearance and meet consumer expectations through well‐designed formulations and products.^10^, ^11^ Like classic retinol, peptides are also one of the most attention‐worthy actives, especially soothing peptides for expression lines that have been reported and popularized 10 years ago.^12^, ^13^ In addition to the reported anti‐wrinkle effects of retinol and neurotransmitter suppressive peptides in Chinese populations, our clinical study examined improvements in skin textures, elasticity, firmness, pores, gloss and stratum corneum hydration. Our study validated the feasibility of using retinol twice daily and supplemented the data and records of retinol‐related efficacy dimensions in the Chinese population.

## 2 MATERIALS AND METHODS

### 2.1 Study design

The 24‐h patch test was carried out at Proya Cosmetics Co. Ltd., Hangzhou, China. Before the study, each participant signed a copy of the informed consent. The protocol of the 24‐h patch test (PCS‐HPT‐21002) was reviewed and approved by the Ethical Commission of Proya Cosmetics Co., Ltd. The 8‐week clinical research was conducted at the SGS Testing Center, Shanghai, China. The research protocol (SHCPCH210302165) was examined and approved by the SGS Ethics Committee for Clinical Research. Benefits, risks and potential complications were explained to the subjects, and informed written consent was obtained from the participants.

### 2.2 Study product

A supramolecular retinol was prepared based on cyclodextrin encapsulation technology as previously described^8^, ^9^ and the preparation process is provided in Supporting Information S1. There were two series of essences containing different concentrations of retinol in the 24‐h patch test. One series of essence contained 0.05%, 0.1%, 0.2%, 0.3% and 0.5% supramolecular retinol; another series of essence contained 0.05%, 0.1%, 0.2%, 0.3% and 0.5% non‐encapsulated retinol. The test product for the 8‐week clinical study was an essence formulated with 0.1% supramolecular retinol and 0.02% acetyl hexapeptide‐1. A 1% supramolecular retinol complex was added to the essence, and the concentration of retinol in the supramolecular retinol complex was 10%; in addition, a 20% acetyl hexapeptide‐1 complex was added to the essence, and the concentration of acetyl hexapeptide‐1 in the acetyl hexapeptide‐1 complex was 0.1%. All these essences for patch tests and clinical studies were prepared using the same formula base (Proya Deep Ocean Energy Wrinkless and Firming Essence). The ingredients in the formula base are provided in Supporting Information S2.

### 2.3 24‐h occlusive patch test

A 24‐h patch test was conducted on 32 subjects using the two series of essence for safety evaluation to detect potential adverse reactions. The test included 32 healthy female and male subjects, aged between 23 and 52 (average aged 32 ± 7). The patches (8‐mm Finn Chambers) with 20 μL essence formula were applied to the upper portion of the subjects' backs and removed after 24 h. Patch test results were interpreted 30 min after removal for immediate urticarial reaction and 48 and 72 h after application for standard patch test reactions, according to the International Contact Dermatitis Research Group recommendations.^14^ Briefly, the condition of the skin in the treated areas was classified as follows: 0 (−) = negative reaction; 1 (±) = doubtful reaction consisting of only faint erythema; 2 (+) = weak positive reaction comprising erythema, infiltration and possibly papules; 3 (++) = strong positive reaction comprising erythema, infiltration, papules and vesicles; 4 (+++) = extreme positive reaction comprising intense erythema and infiltration, as well as coalescing vesicles.

### 2.4 8‐week clinical study

#### 2.4.1 Subjects

Specific subject inclusion and exclusion criteria are provided in Table 1. A total of 33 people were recruited, with one person failing to return for the visit on time, and 32 people (average age 49.5 ± 7.9) having valid data.

**TABLE 1:** Inclusion and exclusion criteria for subject recruitment.

Inclusion criteria	Exclusion criteria
Subjects who were 25–60 years old, with mild photoaged facial skin, large facial pores, visible wrinkles on the facial forehead, eyebrow, canthus, under the eyes, cheeks (fine lines) and nasolabial fold, with clinical score of ≥3 (according to the standard Atlas^15^); Subjects who perceived their face to be loose, dull or rough; stratum corneum hydration of the cheek region was less than 60 C.U. (measured by a Corneometer^®^ CM 825).	Subjects who had externally or internally used retinoids, such as retinol, retinoic acid, tazarotene and adapalene, within 4 months prior to enrolment; Subjects who were pregnant, breast feeding or planning a pregnancy; Subjects who had a history of cosmetic allergies or other serious allergies history; Subjects with systemic diseases or severe skin diseases.

#### 2.4.2 Methods

The subjects were instructed to apply the essence once every other day at first and increase to twice daily after gradually building tolerance and use sunscreens during the day.

Test time points. Subjects were evaluated at baseline and weeks 4 and 8. Before each test, the subjects used amino acid cleansers to clean their faces and wiped them dry with lint‐free paper towels. Then the subjects sat quietly in a standard constant temperature and humidity environment (temperature 20–22°C, humidity 40%–60%) for at least 20 min, and then the dermatological and instrument assessment was conducted by the staff. The dermatological assessment was conducted to evaluate the grade of skin pores. The instrument assessment included the Primos‐CR clinical research system, the Visioscan^®^ VC 20plus for skin topography and the Multi Probe Adapter (MPA) apparatus with probes of Cutometer^®^ MPA580, Corneometer^®^ CM825 and Glossymeter^®^ GL200. During each test, the positioning card was used to locate and measure the same test position.

Wrinkles evaluation methods. Wrinkles levels of cheeks (fine lines), forehead, canthus, nasolabial fold, eyebrow and under the eyes were determined using a 3D roughness analyser Primos‐CR (LMI Technologies, USA) with the two parameters of wrinkle volume and wrinkle length. The smaller the two parameter values, the fewer wrinkles. During the measurement, subjects closed their eyes in a relaxed state.

Skin texture evaluation methods. Visioscan^®^ VC 20plus was used for facial image acquisition and skin texture analysis. The more SEr value is the less rough the skin is. A smaller SEsm value indicates better skin smoothness.

Skin elasticity and firmness evaluation methods. Cutometer^®^ MPA580 (Courage + Khazaka Electronic GMBH, Germany) was applied to measure the skin elasticity and firmness, with the gross elasticity parameter R2, R7 representing the elastic characteristics and F4 representing the firmness of the skin. The closer the R2, R7 parameter is to 1 (100%), the more elastic the skin. The lower the F4 value, the firmer the skin.

Clinical evaluation of skin pores. Dermatological evaluation of the subject's facial pores was carried out according to the standard Atlas. Smaller clinical scores indicate improved pores.

Stratum corneum hydration evaluation methods. The Corneometer^®^ CM825 was used to measure the skin stratum corneum hydration. The higher the measurement value, the higher the stratum corneum hydration.

Skin gloss evaluation methods. Glossymeter^®^ GL200 was used to measure skin gloss. The higher the measurement value, the better the skin gloss.

### 2.5 Statistical analysis

Statistical Package for the Social Sciences was used for data statistics. Chi‐square significance was used to test the results of the patch test, and paired Student *t*‐test method was used to detect the significance of the clinical test data between baseline and the essence‐treated skin. The significance level was *p* ≤ 0.05. Improvement (%) of week 4/week 8 = (test value of week 4/week 8‐baseline value)/baseline value, is used to observe the improvement degree of each parameter. In the analysis results, probability *P*: Chi‐square test or Student *t*‐test, statistically significant with *p* < 0.05; probability close to significant with 0.05 ≤ *p* < 0.10; no statistically significant with *p* ≥ 0.10.

## 3 RESULTS

### 3.1 24‐h occlusive patch test

The 24‐h occlusive patch test result (Table 2) showed (i) only negative reaction or 1 (±) doubtful reactions were observed; (ii) 1 (±) doubtful reactions of both supramolecular retinol and non‐encapsulated retinol increased with the rising retinol concentration; (iii) in the concentration range of 0.05%–0.5%, the supramolecular retinol was more tolerant than non‐encapsulated retinol, with significant difference (*p* < 0.05) or directional significance (0.05 ≤ *p* < 0.10).

**TABLE 2:** Chi‐square significance of supramolecular retinol and non‐encapsulated retinol (*n* = 32).

Retinol concentrations	(±) Doubtful reactions for supramolecular retinol	(±) Doubtful reactions for non‐encapsulated retinol	Probability *p*: Chi‐square test
0.05% retinol	1	4	0.081
0.1% retinol	2	8	0.020
0.2% retinol	5	10	0.070
0.3% retinol	7	12	0.086
0.5% retinol	7	14	0.031

Patch test data suggest the approximate optimal concentration of retinol for topical application in the Chinese population is 0.1%. For a 24‐h occlusive patch test, it is generally considered safe with less than five cases of (±) doubtful reactions among 32 subjects.^16^ There were 1 and 2 cases of (±) doubtful reactions for 0.05% and 0.1% supramolecular retinol, respectively, which were considered safe; while there were no less than five cases of (±) doubtful reactions for 0.2%, 0.3%, 0.5% supramolecular retinol and non‐encapsulated retinol, which were considered as safety risks.

### 3.2 8‐week clinical study

#### 3.2.1 Facial wrinkles

**FIGURE 1:**
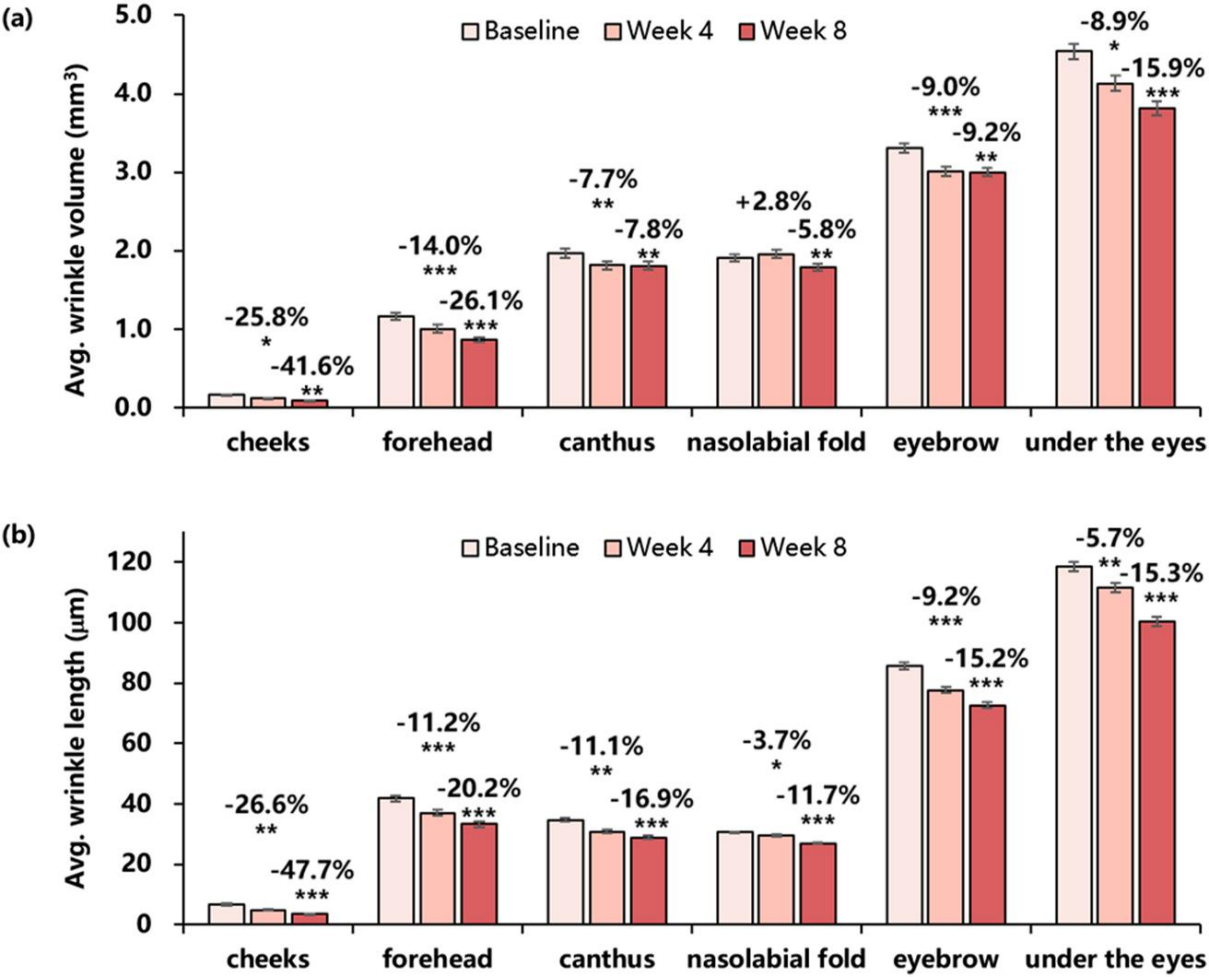
Changes in the wrinkle parameters of cheeks (fine lines), forehead, canthus, nasolabial fold, eyebrow and under the eyes for 8 weeks: (a) wrinkle volume (mean ± SEM), (b) wrinkle length (mean ± SEM). Improvement (%) of week 4/week 8 from baseline is also presented (*P < 0.05 vs. baseline, **P < 0.01 vs. baseline, ***P < 0.001 vs. baseline).

As shown in Figure 1, after an 8‐week application of the essence, the wrinkle parameter of wrinkle volume and wrinkle length of cheeks (fine lines), forehead, canthus, nasolabial fold, eyebrow and under the eyes revealed significant reduction to varying degrees. This indicates that the essence can effectively improve wrinkles all over the face.

**FIGURE 2:**
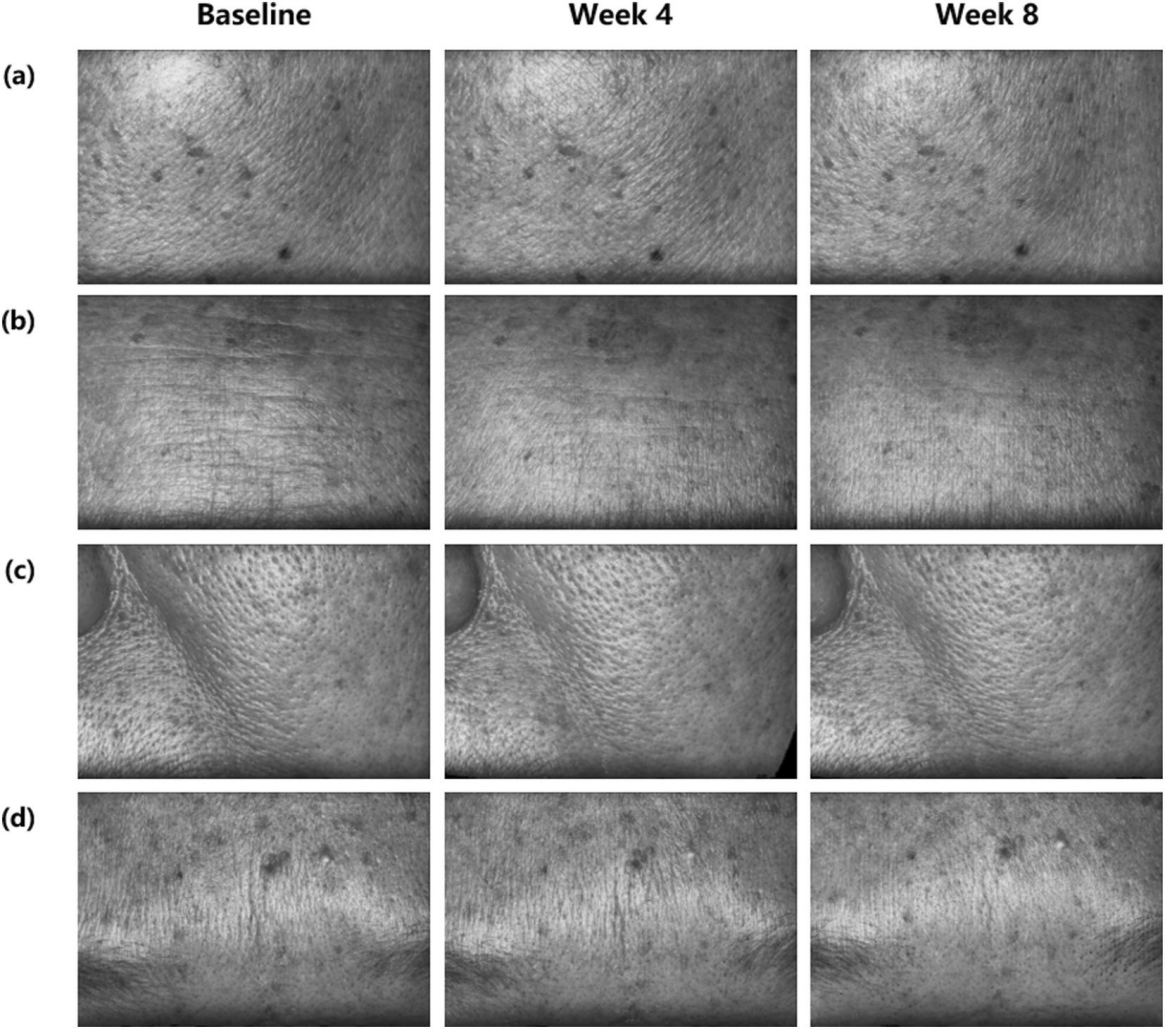
The representative images of the improvement in wrinkles captured by Primos‐CR. These images highlight the impact of the essence on specific facial areas in various subjects: (a) cheeks (fine lines): subject 21, female, 48 years old; (b) forehead: subject 04, female, 49 years old; (c) nasolabial fold: subject 09, female, 52 years old; (d) eyebrow: subject 01, female, 46 years old.

The representative wrinkle improvement of cheeks (fine lines), forehead, nasolabial fold and eyebrow are shown in Figure 2.

#### 3.2.2 Skin textures

**FIGURE 3:**
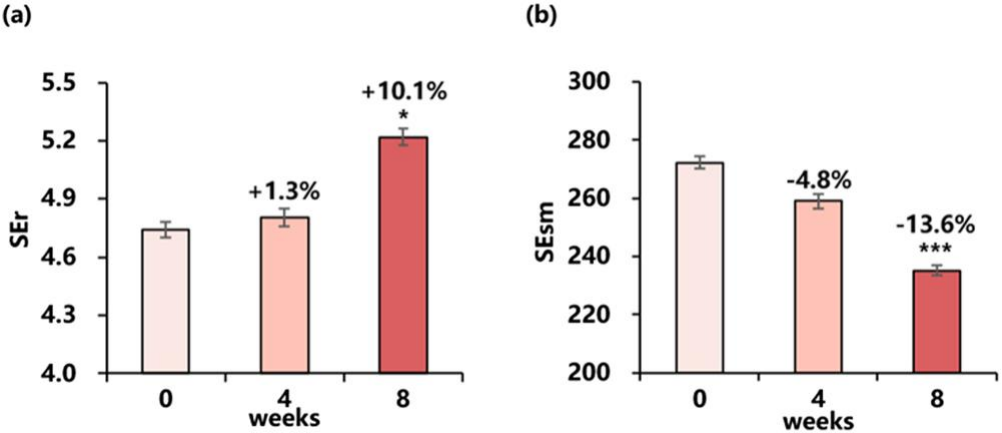
Changes in skin roughness and smoothness for 8 weeks: (a) SEr value (mean ± SEM), (b) SEsm value (mean ± SEM). Improvement (%) of week 4/week 8 from baseline is also presented (*P < 0.05 vs. baseline, ***P < 0.001 vs. baseline).

The clinical tests studied the skin texture of the cheek, and results showed after 8 weeks of essence use, SEr increased by 10.1% and SEsm decreased by 13.6% (Figure 3).

#### 3.2.3 Skin elasticity and firmness

**FIGURE 4:**
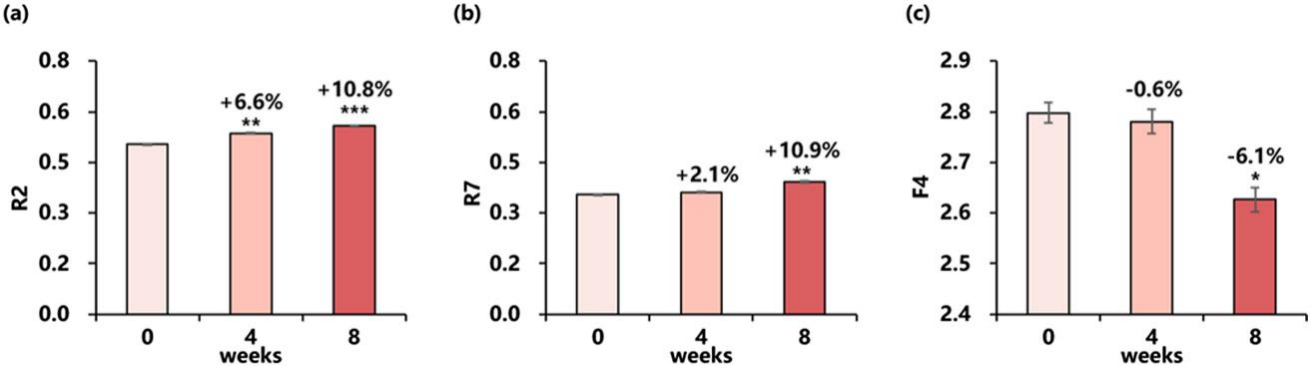
Changes in skin elasticity and firmness for 8 weeks: (a) R2 value (mean ± SEM), (b) R7 value (mean ± SEM), (c) F4 value (mean ± SEM). Improvement (%) of week 4/week 8 from baseline is also presented (*P < 0.05 vs. baseline, **P < 0.01 vs. baseline, ***P < 0.001 vs. baseline).

Compared with baseline, the R2, R7 parameter increased 10.8% and 10.9% respectively, and the F4 value decreased by 6.1% after 8 weeks of use of the essence, revealing the significant improvement of skin elasticity and firmness (Figure 4).

#### 3.2.4 Skin pores

**FIGURE 5:**
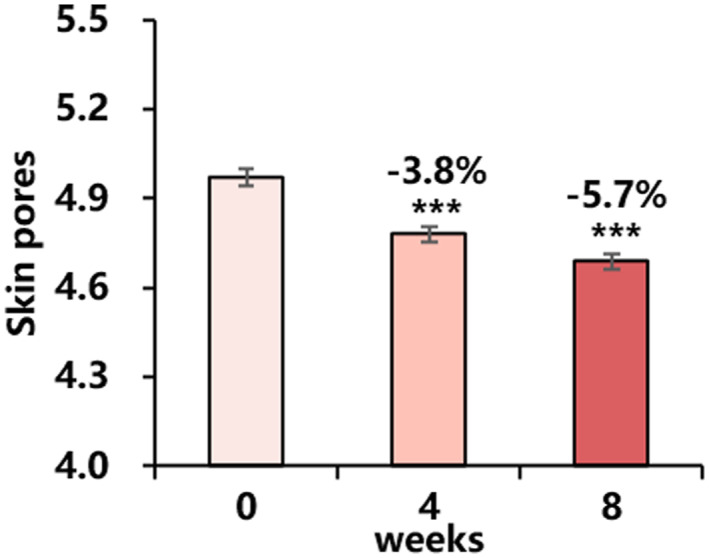
Changes in skin pores by dermatologists' assessment for 8 weeks: skin pores (mean ± SEM). Improvement (%) of week 4/week 8 from baseline is also presented (***P < 0.001 vs. baseline).

Dermatologists' assessment of skin pores showed significant improvement from baseline at each time point. The improvement results showed after 8 weeks of use, the skin pore grade decreased by 5.7% (Figure 5).

#### 3.2.5 Skin gloss

**FIGURE 6:**
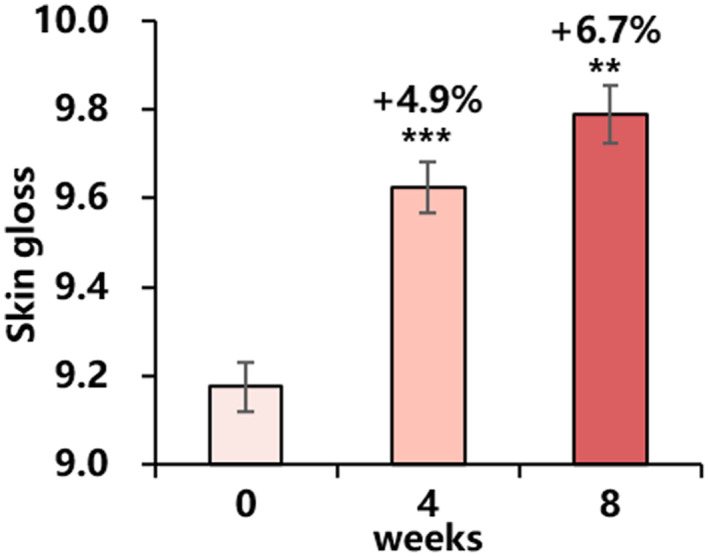
Changes in skin gloss for 8 weeks: skin gloss (mean ± SEM). Improvement (%) of week 4/week 8 from baseline is also presented (**P < 0.01 vs. baseline, ***P < 0.001 vs. baseline).

For Chinese women, loss of gloss was also a phenomenon of skin photoaging. The cheek gloss increased continuously during the use, and the difference was statistically significant before and after use (Figure 6).

#### 3.2.6 Stratum corneum hydration

**FIGURE 7:**
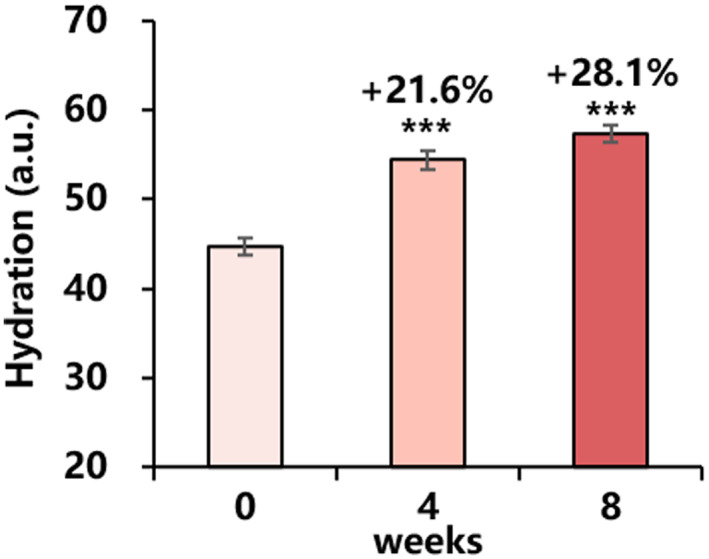
Changes in stratum corneum hydration for 8 weeks: hydration (mean ± SEM). Improvement (%) of week 4/week 8 from baseline is also presented (***P < 0.001 vs. baseline).

Stratum corneum hydration content increased significantly after 4 weeks of use and continued to improve after 8 weeks of continuous use, and the differences were statistically significant before and after use (Figure 7).

#### 3.2.7 Adverse reactions

No adverse reactions were observed during the clinical study, indicating that this product is safe and well tolerated, and is suitable for long‐term use by Chinese people twice daily all over the face.

## 4 DISCUSSION

As China's ageing population grows, there is a growing demand for anti‐ageing products. Retinol is a well‐known and effective anti‐ageing treatment commonly used in skincare products. However, Asians tend to be more intolerant to retinol, which is why it is usually recommended use only once a day at night. The current trend is not to simply increase the concentration of retinol, but to adopt some smart methods.^11^, ^17^ In this study, by employing cyclodextrin encapsulation technology, we were able to decrease the irritation caused by retinol, making it more tolerable for users. Additionally, the formulation was combined with 0.02% acetyl hexapeptide‐1, which adds to the anti‐ageing effects of the product, making it multifunctional and more effective.

Based on our patch test results and other reported clinical studies, 0.1% is the approximate optimal retinol concentration for the general Chinese population, striking a balance between safety and efficacy. Retinol treatment should be administered at concentration that ensures good skin tolerance without causing serious adverse effects like irritation, erythema or dermatitis, which may lead to discontinuation of the therapy. Considering the safety, we have demonstrated that in the concentration range of 0.05%–0.5%, the (±) doubtful reactions in the patch test model increase with the rising retinol concentration. The 24‐h patch test results presented here have shown that there are two cases of (±) doubtful reactions in the 0.1% supramolecular retinol group and eight cases of (±) doubtful reactions in the 0.1% non‐encapsulated retinol group respectively, with significant differences between the two groups. Other studies have shown that retinol can be efficiently encapsulated by cyclodextrins with the benefit of increasing the stability of retinol to light and heat.^18^, ^19^ In this study, supramolecular retinol, formed through the hydrophobic/hydrophilic action of cyclodextrin and the inter‐molecular association of supramolecular association agent, improves the stability and reduces irritation in retinol application. Taken together, our patch test data indicate that the general safe retinol concentration is less than or equal to 0.1%. Therefore, the carefully designed formulation of 0.1% retinol is suitable for anti‐photoaging treatment in the Chinese population. Considering the efficacy, 0.1% is recommended as the approximate optimal concentration of retinol based on other reported clinical studies. When the retinol concentration is less than 0.1%, its efficacy cannot be fully realized. However, concentrations higher than 0.1%, efficacy may not increase, but irritation is more likely to occur. One study shows that in middle‐aged Japanese individuals, 0.04% retinol has less prominent winkle improvements than 0.075% retinol,^4^ suggesting that increasing retinol concentration under 0.1% provides additional benefit. Another study indicates no significant difference in efficacy between retinol concentrations of 0.15% and 0.3%,^2^ suggesting that concentrations higher than 0.15% offer no added benefits.

Facial wrinkles are induced by different mechanisms. In this study, we have used the essence combing 0.1% retinol with 0.02% acetyl hexapeptide‐1, targeting static and dynamic wrinkles, respectively. Retinol has been shown to increase epidermal thickness and upregulate genes for collagen type 1 (COL1A1) and collagen type 3 (COL3A1) with corresponding increases in procollagen I and procollagen III protein expression.^20^ Retinol primarily targets static wrinkles, offering substantial improvement to skin wrinkles. On the other hand, acetyl hexapeptide‐1 targets expression lines and helps regulate muscle contraction to relieve facial expression lines. It acts similarly to topically applied botulinum toxin but is safer and less invasive than botulinum toxin injection for facial aesthetic enhancement. Facial expression lines develop over time as a result of repeated expressions, which reduce skin elasticity and hinder its ability to return to a smooth state, eventually leading to deep and permanent lines.^21^ Overall, our data indicate that the combination of supramolecular retinol and acetyl hexapeptide‐1 effectively addresses both static and dynamic wrinkles while minimising potential irritation. This aligns with the improvements in skin wrinkles, textures, elasticity and firmness observed in the clinical study presented here.

Furthermore, other benefits observed in our clinical study of minimising skin pores and improving skin moisture and gloss are attributed to retinol. Retinol has been shown to promote the proliferation and metabolism of keratinocytes and reduce sebum secretion,^20^, ^22^ which may contribute to the reduction of facial pores. In addition, retinol has been found to increase glycosaminoglycan (GAG) levels in ageing skin.^1^ GAG helps the skin retain moisture at a molecular level, providing hydration and filling in dry areas. The reported mechanism of retinol is likely responsible for the improvements in pore size, skin moisture and gloss observed in the clinical study presented here.

In conclusion, we have found that 0.1% retinol is suitable for twice daily use in the general Chinese population. Our data also indicate the essence containing 0.1% supramolecular retinol and 0.02% acetyl hexapeptide‐1 provides an effective and gentle approach to various photoaging issues for Chinese female facial skin. Our data will hopefully provoke investigators into other cosmeceutical approaches with similar or better efficacy. Furthermore, current clinical studies on topical retinol in the Chinese population mainly focus on the anti‐wrinkle effect.^20^ Data and records on efficacy dimensions of skin gloss, pore fineness, elasticity, firmness, roughness, smoothness and stratum corneum hydration for retinol in Chinese populations are supplemented with our study.

## CONFLICT OF INTEREST STATEMENT

The authors declare no conflicts of interest.

## AUTHOR CONTRIBUTIONS

**Ying Ye**: Conceptualization (Supporting); Data curation (Equal); Investigation (Equal); Methodology (Equal); Writing – original draft (Supporting). **Yanan Li**: Conceptualization (Lead); Data curation (Lead); Investigation (Lead); Methodology (Lead); Writing – original draft (Lead); Writing – review & editing (Lead). **Chenlan Xu**: Data curation (Supporting); Investigation (Supporting); Methodology (Supporting); Writing – original draft (Supporting). **Xiaolan Wei**: Conceptualization (Equal); Data curation (Equal); Investigation (Equal); Methodology (Equal); Writing – review & editing (Equal).

## ETHICS STATEMENT

The 24‐h patch test was carried out at Proya Cosmetics Co. Ltd., Hangzhou, China. Before the study, each participant signed a copy of the informed consent. The protocol of the 24‐h patch test (PCS‐HPT‐21002) was reviewed and approved by the Ethical Commission of Proya Cosmetics Co., Ltd. The 8‐week clinical research was conducted at the SGS Testing Center, Shanghai, China. The research protocol (SHCPCH210302165) was examined and approved by the SGS Ethics Committee for Clinical Research. Benefits, risks and potential complications were explained to the subjects, and informed written consent was obtained from the participants.

## Supporting information

Supplementary Material

## Data Availability

The data that support the findings of this study are available from the corresponding author upon reasonable request.
