# Supplementary material for: Improvement of mild photoaged facial skin in middle‐aged Chinese females by a supramolecular retinol plus acetyl hexapeptide‐1 containing essence
Source: Skin Health Dis. 2023 May 7;3(4):e239. doi: 10.1002/ski2.239 (PMC10395635; doi:10.1002/ski2.239)
Supplement: Supplementary file 1 — Supplementary Material [file SKI2-3-e239-s001.docx]

Improvement of mild photoaged facial skin in middle-aged Chinese females by a supramolecular retinol plus acetyl hexapeptide-1 containing essence

Ying Ye, Yanan Li*, Chenlan Xu, Xiaolan Wei

Research & Innovation Center, Proya Cosmetics Co. Ltd., Hangzhou, China

**Supplementary information**

S1. The preparation process of cyclodextrin encapsulation technology.

In brief, firstly retinol was encapsulated through cavitation in hydroxypropyl γ-cyclodextrin, which had a macrocyclic molecular structure that was internally hydrophobic and externally hydrophilic. Then the associating agent (composed of hydroxypropyl methylcellulose stearoxy ether and PEG/PPG/polybutylene glycol-8/5/3 glycerin) and the hydrophilic hydroxyl group of the outer cavity of the hydroxypropyl γ-cyclodextrin associated through intermolecular force to form a stable supramolecular retinol structure (the retinol concentration in the supramolecular retinol structure was 10%).

S2. The ingredients contained within the formula base were:

Aqua, Cyclopentasiloxane, Dipropylene Glycol, Propanediol, Tridecyl Isononanoate, Phytosteryl/Octyldodecyl Lauroyl Glutamate, Glycerin, Acetyl Glucosamine, Hydroxyphenyl Propamidobenzoic Acid, Acetyl Tetrapeptide-2, Glycine Soja (Soybean) Extract, Niacinamide, Stephania Tetrandra Extract, Dipotassium Glycyrrhizate, Hydrogenated Phosphatidylcholine, Dipalmitoyl Hydroxyproline, Butyrospermum Parkii (Shea) Butter, Squalane, Ceramide NP, Glycerophosphoinositol Choline, Panthenol, Hydrolyzed Rhodophyceae Extract, Allantoin, Theanine, 4-t-Butylcyclohexanol, Sodium Acetylated Hyaluronate, Sodium Hyaluronate, Sodium Hyaluronate Crosspolymer, Hydrolyzed Sodium Hyaluronate, Mauritia Flexuosa Fruit Oil, Adenosine, Superoxide Dismutase, Mevalonolactone, Soluble Proteoglycan, Olea Europaea (Olive) Leaf Extract, Calcium Pantetheine Sulfonate, Maris Aqua, Trehalose, Caprylyl Glycol, Butylene Glycol, Pentylene Glycol, Phytosteryl/Behenyl/Octyldodecyl Lauroyl Glutamate, Hydrogenated Lecithin, Caprylic/Capric Triglyceride, Pentaerythrityl Tetraethylhexanoate, Sorbitan Stearate, Pentaerythrityl Tetra-Di-t-Butyl Hydroxyhydrocinnamate, Phenyl Trimethicone, Dimethicone Crosspolymer, Polyglyceryl-10 Stearate, Polyglyceryl-3 Methylglucose Distearate, Polysorbate 60, Isostearyl Glyceryl Ether, Boron Nitride, Ammonium Acryloyldimethyltaurate/VP Copolymer, Hydroxyacetophenone, Citric Acid, 1,2-Hexanediol, Ethylhexylglycerin, Xanthan Gum, Disodium Edta, Phenethyl Alcohol, Benzotriazolyl Dodecyl p-Cresol, Phenoxyethanol, Caramel, Parfum.
